# Candidate biomarkers for treatment benefit from sunitinib in patients with advanced renal cell carcinoma using mass spectrometry-based (phospho)proteomics

**DOI:** 10.1186/s12014-023-09437-6

**Published:** 2023-11-08

**Authors:** Hanneke van der Wijngaart, Robin Beekhof, Jaco C. Knol, Alex A. Henneman, Richard de Goeij-de Haas, Sander R. Piersma, Thang V. Pham, Connie R. Jimenez, Henk M. W. Verheul, Mariette Labots

**Affiliations:** 1 Department of Medical Oncology, Cancer Center Amsterdam, Amsterdam UMC, Vrije Universiteit Amsterdam, De Boelelaan 1117, 1081 HV Amsterdam, The Netherlands; 2 Department of Medical Oncology, Erasmus University Medical Center, Rotterdam, The Netherlands

**Keywords:** Cancer, Mass spectrometry-based phosphoproteomics, Tyrosine kinase inhibitors, Renal cell carcinoma, Sunitinib

## Abstract

The tyrosine kinase inhibitor sunitinib is an effective first-line treatment for patients with advanced renal cell carcinoma (RCC). Hypothesizing that a functional read-out by mass spectrometry-based (phospho, *p-*)proteomics will identify predictive biomarkers for treatment outcome of sunitinib, tumor tissues of 26 RCC patients were analyzed. Eight patients had primary resistant (RES) and 18 sensitive (SENS) RCC. A 78 phosphosite signature (p < 0.05, fold-change > 2) was identified; 22 *p*-sites were upregulated in RES (unique in RES: BCAR3, NOP58, EIF4A2, GDI1) and 56 in SENS (35 unique). EIF4A1/EIF4A2 were differentially expressed in RES at the (*p-*)proteome and, in an independent cohort, transcriptome level. Inferred kinase activity of MAPK3 (p = 0.026) and EGFR (p = 0.045) as determined by INKA was higher in SENS. Posttranslational modifications signature enrichment analysis showed that different *p*-site-centric signatures were enriched (p < 0.05), of which FGF1 and prolactin pathways in RES and, in SENS, vanadate and thrombin treatment pathways, were most significant. In conclusion, the RCC (phospho)proteome revealed differential *p-*sites and kinase activities associated with sunitinib resistance and sensitivity. Independent validation is warranted to develop an assay for upfront identification of patients who are intrinsically resistant to sunitinib.

**Supplementary Information:**

The online version contains supplementary material available at 10.1186/s12014-023-09437-6.

## Background

The treatment landscape in metastatic renal cell carcinoma (mRCC) has changed dramatically in the past 15 years. Anti-angiogenic tyrosine kinase inhibitors (TKIs), such as sunitinib, sorafenib, axitinib, pazopanib and cabozantinib, are an effective treatment option for patients with mRCC. Since their introduction, the median overall survival (OS) has improved from 15–17 months before 2004 [1–4] to 23–29 months with TKI monotherapy [5–7]. Combining TKI’s with immune checkpoint inhibitors (ICI) has further improved the 12-month overall survival rate from 72% [8] to 90% [9, 10]. With the vast expansion of therapeutic options, optimization of treatment selection strategies for individual patients becomes more important. Sunitinib is an oral multi-targeted TKI targeting mainly the Vascular Endothelial Growth Factor Receptors (VEGFR 1 and 2), Platelet-Derived Growth Factor Receptors (PDGFR-alpha and PDGFR-beta) and stem cell factor receptor (KIT), though many off-target effects are observed [11]. Patients receiving first-line treatment with sunitinib have a median progression free survival (PFS) of 8.4—11 months, with an objective response rate of 25—47% [7, 12]. However, all patients eventually relapse due to acquired resistance, and 13–29% does not benefit from treatment at all [12–14]. Moreover, up to 53% of patients require dose interruptions and in 12% therapy is discontinued because of adverse events [12]. Sunitinib remains one of the preferred first-line treatment options for patients with favorable-risk clear cell RCC (ccRCC) and non-ccRCC [15–17]. To improve treatment benefit from sunitinib, a predictive biomarker would be of significant clinical value.

Tissue-based baseline predictive biomarkers for sunitinib in RCC are lacking. Although a large number of candidate molecular biomarkers have been under investigation, none have been prospectively validated [18]. Thus far, most attempts have applied immunohistochemistry, panel DNA or RNA sequencing and PCR for target detection [19]. However, due to multiple resistance mechanisms in RCC, characteristically driven by a multitude of aberrantly activated kinase signaling pathways [20] instead of a single oncogenic driver mutation, genomics-based analysis alone is most likely not sufficient to predict response to sunitinib [21]. A functional pathway analysis may be a more promising approach [22, 23].

Proteins are the driving force of cellular function, including intracellular signaling and immune responses. Post-translational modifications, such as phosphorylation, have a major role in regulation of protein function and activity. (Phospho)proteomics based on liquid chromatography coupled to tandem mass spectrometry (LC–MS/MS) offers insight in aberrantly activated kinase signaling pathways and potential drug targets through the global analysis of phosphorylated proteins. This method has high potential for patient stratification and prediction of therapy response [24–28]. In particular, phosphotyrosine-(pTyr)-phosphoproteomics provides an opportunity for the identification of patient subgroups likely to benefit from TKI’s [29]. As only 1% of all protein phosphorylations occur on tyrosine residues [30], enrichment of tyrosine phosphorylated peptides is necessary prior to LC–MS/MS.

We here aimed to identify baseline tissue-based molecular biomarkers for prediction of (lack of) treatment benefit to sunitinib in patients with advanced RCC, using MS-based pTyr-phosphoproteomics and global expression proteomics.

## Materials and methods

### Patient selection

From the hospital pathology database, patients with RCC were selected who had undergone tumor nephrectomy or metastasectomy between 2000 and 2013, and thereafter received palliative treatment with sunitinib in the Amsterdam University Medical Centers (Amsterdam UMC), location VUmc. Clinical data were collected retrospectively from the hospital case records. Patients were classified as “sensitive” if they had PFS ≥ 12 weeks and radiological stable disease or objective response, or “primary resistant” if they exhibited radiological progressive disease at first evaluation (PFS < 12 weeks). Since archival tissue was used for the purpose of scientific research, and collected within the context of routine clinical practice procedures, the Dutch Medical Research Involving Human Subjects Act does not apply. Patients treated at Amsterdam UMC had the possibility to opt-out for the use of their data and tissue for research purposes.

### Tumor tissue collection and sample processing for LC–MS/MS

Frozen pre-treatment tumor resection specimens, acquired through standard care procedures and stored at − 80 °C, were collected from the hospital biobank. The tumor samples were cut (Leica CM1850) in 10-µm cryosections at – 20 °C, transferred to precooled 1.5-ml Eppendorf vials and stored at – 80 °C. Lysis was performed using approximately 1 ml 9 M urea buffer per sample, followed by 1 min vortexing (maximum speed), sonication (18-μm amplitude) and centrifugation (15 min, maximum speed). The cleared lysate was aliquotted and stored at − 80 °C until further use. The BCA protein assay (ThermoPierce, Rockford, IL) was used to determine protein concentration. Cell lysates were reduced in 4 mMDTT for 20 min at 60 °C, cooled to room temperature, and subsequently alkylated in 10mMiodoacetamide for 15 min in the dark. After dilution to 2 M urea using 20 mM HEPES buffer pH 8.0, the lysate was digested with 20 μg Sequencing Grade Modified trypsin/(Promega, Leiden, The Netherlands) per mg protein by overnight incubation at 22 °C. Digestion was then stopped by adding trifluoroacetic acid (TFA) to a final concentration of 1%. Samples were incubated for 15 min on ice, centrifuged for 5 min at 1800 × g, and transferred to a new tube. Tryptic digests were desalted using 1-ml Oasis HLB cartridges (Waters, Milford, MA). After pre-wetting with acetonitrile (ACN) and equilibration of the column with 0.1% TFA, peptides were loaded. The column was washed using 0.1% TFA before elution into glass vials with 40% ACN/0.1% TFA. Eluates were lyophilized for 48 h and stored at − 80 °C until further use.

### Control samples

As quality control samples, the colorectal cancer cell line HCT116 and a reference sample of tissue-mixture (containing pooled lysates of tumor samples of colorectal cancer, melanoma, non-small cell lung cancer and hepatocellular carcinoma) were used. HCT116 cells were obtained from the American Type Culture Collection. Cells were cultured in Dulbecco’s Modified Eagle Medium (DMEM), supplemented with 10% fetal bovine serum (FBS), 100 U/ml sodium penicillin and 100 µg/ml streptomycin, and maintained at 37 °C. Plated cells were washed twice with phosphate-buffered saline (PBS) and lysed using 9M urea buffer. Cells were scraped and the lysate was sonicated and centrifuged for 15 min at maximum speed. Aliquots of lysate were stored at − 80 °C. Further processing was done as described before.

### Immunoprecipitation and protein identification

Tumor samples were processed in 3 batches, each containing samples from patients with sensitive and resistant tumors. Immunoprecipitation (IP) of tyrosine phospho-peptides was performed using the PTMScan kit (P-Tyr-1000) from Cell Signaling Technology (Leiden, The Netherlands) as described elsewhere [32, 34]. Briefly, lyophilized phosphopeptides were dissolved in IAP buffer (20 mM Tris–HCl pH 7.2, 10 mM sodium phosphate and 50 mM NaCl) and incubated with 2 μl P-Tyr-1000 beads per mg protein at 4 °C for 2 h. After washing in cold IAP buffer and Milli-Q water, peptides were eluted from the beads in two steps in 0.15% TFA, desalted in 20 μl Proxeon Stage Tips (Thermo Scientific) using 0.1% TFA, eluted with 80% ACN/0.1% TFA into LC autosampler vials, and stored at 4 °C until LC–MS/MS measurement on the same day. Peptides were separated on a pepmap Acclaim column (75 μm ID × 500 mm, 1.9 μm C18) connected to a pepmap Acclaim trap column (75 μm ID × 10 mm 3 μm C18) and running at 300 nl/min as described elsewhere [32, 33] on an Ultimate 3000 nanoLC- (Dionex LC-Packings, Amsterdam, The Netherlands) connected to a Q Exactive mass spectrometer (Thermo Fisher, Bremen, Germany) using a 2 h gradient (8–32% acetonitrile in 0.1% formic acid). Intact masses were measured at resolution 70,000 (at *m/z* 200) in the Orbitrap analyser using an AGC target value of 3E6 charges. The top 10 peptide signals (charge-states 2 + and higher) were submitted to MS/MS in the HCD (higher-energy collision) cell (1.4 u-amu isolation width, 25% normalized collision energy). MS/MS spectra were acquired at resolution 17.500 (at m/z 200) in the Orbitrap using an AGC target value of 1E6 charges, MaxIT of 80 ms and an underfill ratio of 0.1%. Dynamic exclusion was applied with a repeat count of 1 and an exclusion time of 30 s.

LC–MS/MS spectra were searched against the Uniprot human reference proteome FASTA file (release August 2015, 62447 entries, no fragments) using MaxQuant 1.5.2.8 [35]. Enzyme specificity was set to trypsin and up to two missed cleavages were allowed. Cysteine carboxamidomethylation (Cys, + 57.021464 Da) was treated as fixed modification and serine, threonine and tyrosine phosphorylation (+ 79.966330 Da), methionine oxidation (Met, + 15.994915 Da) and N-terminal acetylation (N-terminal, + 42.010565 Da) as variable modifications. Peptide precursor ions were searched with a maximum mass deviation of 4.5 ppm and fragment ions with a maximum mass deviation of 20 ppm. Peptide, protein and site identifications were filtered at a false discovery rate (FDR) of 1% using the decoy database strategy. The minimal peptide length was 7 amino acids and the minimum Andromeda score for modified peptides was 40, with the corresponding minimum delta score set at 17 [36]. Proteins that could not be differentiated based on MS/MS spectra alone were grouped into protein groups (default MaxQuant settings). (Phospho)peptide identifications were propagated across samples using the match-between-runs option checked. Searches were performed with the label-free quantification option selected. A normalization factor derived from the total count of matched protein lysates was applied to scale peptide intensities for each pTyr capture.

### Protein expression profiling

Protein lysates (50 μg) were separated on precast 4–12% gradient gels using the NuPAGE SDS‐PAGE system (Invitrogen, Carlsbad, CA). Following electrophoresis, gels were fixed in 50% ethanol/3% phosphoric acid solution and stained with Coomassie R‐250. Gel lanes were cut into five bands, and each band was cut into ~ 1 mm3 cubes. Gel cubes were washed with 50 mM ammonium bicarbonate/50% acetonitrile and were transferred to a 1.5 ml microcentrifuge tube, vortexed in 400 μl 50 mM ammonium bicarbonate for 10 min, and pelleted. The supernatant was removed, and the gel cubes were vortexed in 400 μl 50 mM ammonium bicarbonate/50% acetonitrile for 10 min. After pelleting and removal of the supernatant, this wash step was repeated. Subsequently, gel cubes were reduced in 50 mM ammonium bicarbonate supplemented with 10 mM DTT at 56 °C for 1 h. The supernatant was removed, and gel cubes were alkylated in 50 mM ammonium bicarbonate supplemented with 50 mM iodoacetamide for 45 min at room temperature in the dark. Next, gel cubes were washed with 50 mM ammonium bicarbonate/50% acetonitrile dried in a vacuum centrifuge at 50 °C for 10 min and covered with trypsin solution (6.25 ng/μl in 50 mM ammonium bicarbonate). Following rehydration with trypsin solution and removing excess trypsin, gel cubes were covered with 50 mM ammonium bicarbonate and incubated overnight at 25 °C. Peptides were extracted from the gel cubes with 100 μl of 1% formic acid (once) and 100 μl of 5% formic acid/50% acetonitrile (twice). For each sample the three extracts were pooled and stored at – 20 °C until use. Before LC‐MS, the extracts were concentrated in a vacuum centrifuge at 50 °C, and volumes were adjusted to 50 μl by adding 0.05% formic acid, filtered through a 0.45 μm spin filter, and transferred to an LC autosampler vial.

### Statistical analysis and biological pathway analysis

Cluster analysis of phosphopeptides and phosphosites was performed using hierarchical clustering. Phosphopeptide intensities were normalized to zero mean and unit variance for each phosphopeptide. Normalization of phosphopeptide intensities and cluster analyses were performed in R version 3.5.1. For comparative analyses, only high confidence class 1 phosphosites were considered. Aiming to distinguish a phosphosite and protein signature predictive of treatment outcome of sunitinib, differential expression patterns were analyzed using the Linear Models for Microarray and RNA-Seq Data (limma) package version 3.36.5 for R [37, 38] (filters: p < 0.05, fold change (FC) > 2, ≥ 30% data presence, i.e. there must be a non-zero value in at least 30% of samples in the group with highest abundance). Differential expression of proteins was analyzed using the filters: p < 0.05, FC > 2 and ≥ 50% data presence; here, with a more complete data matrix, a stricter filter could be applied. No imputation of data was performed. Heatmap visualization and hierarchical clustering was done with the R package ComplexHeatmap version 2.2.0 [39]. Differential proteins were imported into Cytoscape version 3.5 [40], and gene ontology analysis was performed in Cytoscape with the BiNGO app version 3.0.3 [41], using ontology and organism annotation definitions downloaded on 8 July 2019 via http://geneontology.org.

### Kinase activity analysis

Per sample, a ranking of most activated kinases was generated using the Integrative Inferred Kinase Activity (INKA) data analysis pipeline [24], taking both information on phosphorylated kinases and their substrates into account. Differentially activated kinases were identified and level of significance was determined by Mann–Whitney U-test.

### Post-translational modifications signature enrichment analysis (PTM-SEA)

PTM-SEA [42] was performed using the Phospho (STY).txt Max Quant search result file after filtering out decoy and contaminant site entries, to identify site-specific signatures of kinase activities and signaling pathways, overrepresented in each of the 2 groups. Phosphosites were ranked using − 10 * sign(logFC) * log10(P-Value) as a measure, where the P-value and logFC were calculated in a differential analysis by limma version 3.38.3. and used as inputs to run the PTM-SEA algorithm in GenePattern [43] (https://cloud.genepattern.org). The PTM signature sets were those defined in PTMsigDB v1.9.0 (human, flanking sequence format, file ptm.sig.db.all.flanking.human.v1.9.0.gmt) downloaded from https://github.com/broadinstitute/ssGSEA2.0. Results were visualized in R. Significantly enriched signatures were reported (FDR < 0.25).

### Exploration of (phospho)proteomics candidates in transcriptome data of an independent cohort

Publicly available transcriptomics data from an independent cohort previously described by Beuselinck et al [44] was used. CEL files containing Affymetrix array signals from 59 patients with ccRCC, treated with sunitinib, were obtained and processed in R (package “oligo”). Group comparison analysis was done in R (package “LIMMA”). All significantly (p < 0.05) differentially expressed transcripts were considered. Expression levels of differentially expressed proteins from our proteomics analysis (p < 0.05 & FC > 2 & ≥ 50% data points in the highest group) were compared to the expression of matching transcripts in the validation cohort at gene level, the percentage of overlapping proteins/transcripts was reported.

### Data and materials availability

The mass spectrometry proteomics data have been deposited to the ProteomeXchange Consortium via the PRIDE [45] partner repository with the dataset identifier PXD043514.

## Results

### Clinicopathological characteristics

Twenty-six patients with mRCC were identified who underwent resection of a primary tumor (*n* = 23) or metastatic lesion (*n* = 3) and received sunitinib as first-line palliative therapy upon progression or relapse (Table 1, Additional file 6: Table S1). The median time between surgery and start of sunitinib was six months (range 1–63). Eighteen patients were sensitive to sunitinib, of whom six had an objective response. The median PFS (mPFS) in this group was 8.8 months (range 5–62.3). Eight patients had progressive disease as best response (mPFS 2.3 months, range 1.5–2.8).

**Table 1:** Patient characteristics

Variable	All patients (*n* = 26)	Sensitive (*n* = 18)	Primary resistant (*n* = 8)
Age (years), median (range)	60 (20–80)	61 (40–79)	58 (20–80)
Sex, *n* (%)			
Female	11 (42)	8 (44)	3 (38)
Male	15 (58)	10 (56)	5 (62)
Histology, *n* (%)			
Clear cell carcinoma	17 (65)	13 (72)	4 (50)
Papillary carcinoma	3 (12)	1 (6)	2 (25)
Mixed type^1^	6 (23)	4 (22)	2 (25)
Prior systemic therapy, *n* (%)			
0	17 (65)	10 (55)	7 (88)
1	8 (31)	7 (39)	1 (12)
2	1 (4)	1 (6)	–
PFS (months), median (range)		8.8 (5–62.3)	2.3 (1.5–2.8)
Time to sunitinib (months), median (range)	6 (1–63)	6 (1–63)	6 (1–24)

^1^ Consists of more than one histological type: clear cell + papillary, clear cell + sarcomatoid, clear cell + eosinophilic variant. Time to sunitinib indicates interval between resection and initiation of sunitinib treatment; PFS, progression-free survival

### Tyrosine-phosphoproteomics analysis

**Fig. 1:**
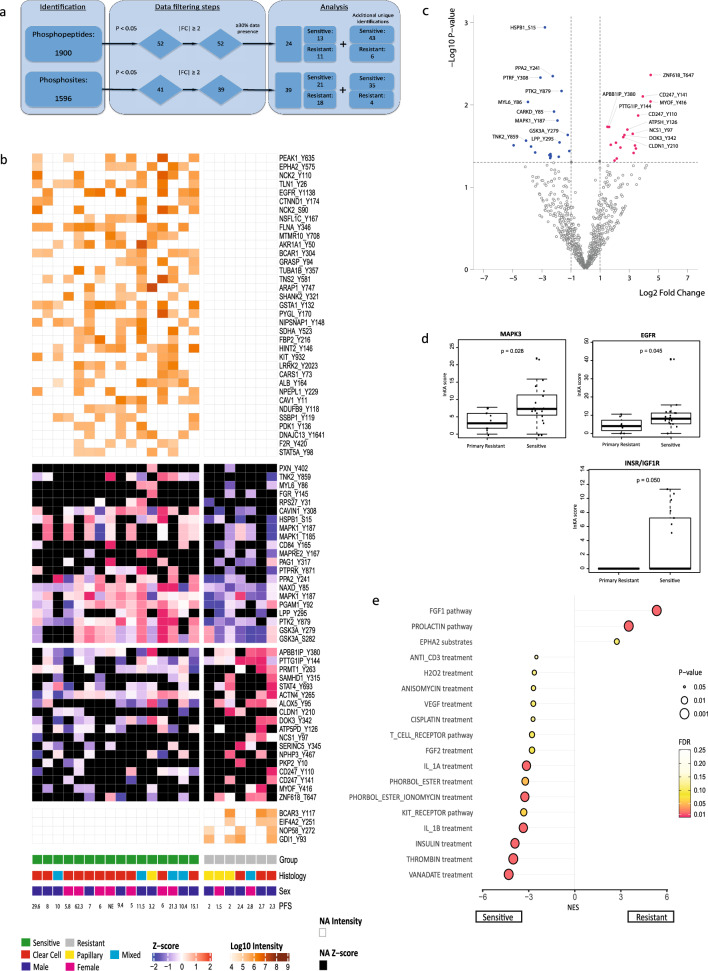
Phosphoproteome analysis of patients with RCC sensitive or resistant to sunitinib. **a** Overview of the data filtering steps applied in phosphosite and phosphopeptide analysis, including the effect of each filter on the total number. **b** Heatmap of the differentially detected phosphosites (*n* = 78) in sensitive and primary resistant patients, split by group. The heatmap is a concatenation of 3 heatmaps created with R package ComplexHeatmap. The first and third heatmaps were created with log10-transformed intensity values for phosphosites that were uniquely identified (“exclusive”) in the sensitive resp resistant patient group and had a data presence of at least 30%. The second heatmap was created with log10-transformed intensity values for significantly differential phosphosites (“non-exclusive”; p, 0.05, FC ≥ 2). This heatmap was clustered by columns but not by rows. Instead, rows were sorted by fold change and split by the sign of the fold change (down-regulated phosphosites in the upper part, up-regulated phosphosites in the lower part). Column splitting was at the first split of the column clustering dendrogram, and dendrogram plotting was set to FALSE. The column ordering in the resulting concatenated heatmap was determined by the middle heatmap. No imputation of data is performed. Euclidean distance and Ward’s linkage method were used. Black squares indicate non-identified phosphosites in this subgroup. Histology = histological subtype as determined by pathologist review; PFS = progression free survival in months; NE = not evaluable. **c** Volcano plot of for statistical comparison of differential class 1 phosphosites between the Sensitive and Resistant groups were generated in R with the ggplot2 package. The top 10 significant phosphosites for each group are indicated by labeling. Labels are given for the phosphosite, not the specific type of phosphopeptide in which it was detected. **d** Boxplots of differentially activated kinases based on INKA analysis. P-values by Mann–Whitney U-test. X-axis: 2 groups (primary resistant versus sensitive patients). Y-axis: INKA score of the kinase, based on kinase- and substrate-centric analyses. **e** PTM-SEA identified site-specific signatures of kinase activities and signaling pathways, overrepresented in each of the 2 groups. Phosphosites were ranked using the quantity -10 * sign(logFC) * log10(P-Value), where the P-value and logFC were calculated in a differential analysis by limma and used as inputs to the 20161013 version of ssGSEA2.0.R. The PTM-sets were defined in ptm.sig.db.all.flanking.human.v1.9.0.gmt. Significantly enriched signatures are presented in this figure (p < 0.05). X-axis represents the enrichment score (negative score = enriched in sensitive patients, positive score = enriched in resistant patients)

Twenty-three out of 26 tumor tissues (16 sensitive and seven primary resistant patients) were evaluable for tyrosine-phosphoproteomics, with a median protein input of 5 mg (range 2–5 mg) per sample. Three samples were considered not evaluable; two had a very low phosphopeptide yield and one had a low protein yield, hindering lysate-based normalization. In total, 2656 unique class 1 phosphosites were identified in tumor and control samples. After eliminating all control sample-specific sites, 1596 unique class 1 phosphosites remained for further comparative analysis between the two groups (86% tyrosine, 9% serine and 5% threonine, showing adequate enrichment for tyrosine phosphorylated peptides), with a median of 415 (range 266–713) phosphosites per sample. Identified and quantified phosphosites and phosphopeptides are presented in Additional file 7: Table S2 and Additional file 8: Table S3. The primary analysis, aiming to identify markers distinguishing sensitive from resistant patients, was performed on phosphosite data. Unsupervised cluster analysis of all identified phosphosites could not separate sensitive from resistant patients (Additional file 1: Fig. S1a). After data filtering (p < 0.05, FC > 2) (Fig. 1a), a signature of 78 differential phosphosites was identified, comprising 22 upregulated sites in resistant patients; 4 of these were uniquely identified in resistant patients (BCAR3, NOP58, EIF4A2 and GDI1, filtered for ≥ 30% data presence in the group with highest abundance). Fifty-six phosphosites of aforementioned signature were upregulated in sensitive patients; 35 of these were uniquely identified in this subgroup (Table 2). This selection of most differential phosphosites split by group is shown in Fig. 1b. Top-10 differential phosphosites in each group are shown in Fig. 1c. Phosphopeptide clustering data are available in Additional file 2: Fig. S2a, b.

**Table 2:** Candidate phosphosite signature (n = 78) for prediction of sunitinib treatment outcome in RCC

	Phosphosite	p-value	FC
Phosphosites upregulated in primary resistant patients			
Uniquely upregulated in resistant tumors	BCAR3_Y117	n/a	n/a
	EIF4A2_Y251	n/a	n/a
	NOP58_Y272	n/a	n/a
	GDI1_Y93	n/a	n/a
Differentially upregulated (not unique)	ZNF618_T647	0.004	22.2
	CD247_Y141	0.008	15.2
	MYOF_Y416	0.009	22.0
	CD247_Y110	0.013	12.2
	APBB1IP_Y380	0.018	2.8
	PTTG1IP_Y144	0.018	3.1
	ATP5PD_Y126	0.020	7.3
	NCS1_Y97	0.022	9.4
	DOK3_Y342	0.023	6.3
	CLDN1_Y210	0.025	6.0
	STAT4_Y693	0.029	4.2
	PRMT1_Y263	0.030	3.3
	NPHP3_Y467	0.031	10.5
	ALOX5_Y95	0.033	5.3
	PKP2_Y10	0.034	11.0
	SERINC5_Y345	0.038	9.8
	ACTN4_Y265	0.045	4.4
	SAMHD1_Y315	0.047	3.9
Phosphosites upregulated in sensitive patients			
Uniquely upregulated in sensitive tumors	PEAK1_Y635	n/a	n/a
	EPHA2_Y575	n/a	n/a
	NCK2_Y110	n/a	n/a
	TLN1_Y26	n/a	n/a
	EGFR_Y1138	n/a	n/a
	CTNND1_Y174	n/a	n/a
	CDK2_S90	n/a	n/a
	NSFL1C_Y167	n/a	n/a
	FLNA_Y346	n/a	n/a
	MTMR10_Y708	n/a	n/a
	AKR1A1_Y50	n/a	n/a
	BCAR1_Y304	n/a	n/a
	GRASP_Y94	n/a	n/a
	TUBA1B_Y357	n/a	n/a
	TNS2_Y581	n/a	n/a
	ARAP1_Y747	n/a	n/a
	SHANK2_Y321	n/a	n/a
	GSTA1_Y132	n/a	n/a
	PYGL_Y170	n/a	n/a
	NIPSNAP1_Y148	n/a	n/a
	SDHA_Y523	n/a	n/a
	FBP2_Y216	n/a	n/a
	HINT2_Y146	n/a	n/a
	KIT_Y932	n/a	n/a
	LRRK2_Y2023	n/a	n/a
	CARS1_Y73	n/a	n/a
	ALB_Y164	n/a	n/a
	NPEPL_Y229	n/a	n/a
	CAV1_Y11	n/a	n/a
	NDUFB9_Y118	n/a	n/a
	SSBP1_Y119	n/a	n/a
	PDK1_Y136	n/a	n/a
	DNAJC13_Y1641	n/a	n/a
	F2R_Y420	n/a	n/a
	STAT5A_Y98	n/a	n/a
Differentially upregulated (not unique)	HSPB1_S15	0.001	− 6.9
	PPA2_Y241	0.004	− 4.8
	CAVIN1_Y308	0.005	− 8.6
	PTK2_Y879	0.007	− 3.1
	MYL6_Y86	0.009	− 15.5
	NAXD_Y85	0.012	− 4.5
	MAPK1_Y187	0.015	− 3.8
	GSK3A_Y279	0.023	− 2.3
	TNK2_Y859	0.027	− 16.9
	LPP_Y295	0.028	− 3.4
	PXN_Y402	0.031	− 30.6
	FGR_Y145	0.032	− 13.4
	GSK3A_S282	0.036	− 2.2
	RPS27_Y31	0.038	− 11.0
	MAPRE2_Y167	0.040	− 5.3
	MAPK1_Y187	0.041	− 5.6
	MAPK1_T185	0.041	− 5.6
	PAG1_Y317	0.042	− 5.3
	PTPRK_Y871	0.042	− 5.3
	PGAM1_Y92	0.042	− 3.6
	CD84_Y165	0.044	− 5.4

The 22 phosphosites upregulated in resistant patients, 4 of which were uniquely identified in this group, were linked to various immune processes by gene ontology analysis, such as response to interleukin-18, immune response and immune effector process. The 56 phosphosites upregulated in sensitive patients (of which 35 uniquely identified) were linked to various cellular regulatory and signaling processes, such as enzyme linked receptor protein- and transmembrane receptor protein tyrosine kinase signaling pathways, peptidyl-tyrosine autophosphorylation, positive regulation of cell motility and VEGFR and Epidermal Growth Factor Receptor (EGFR) signaling pathways (Additional file 3: Fig. S3). Additional file 9: Table S4 lists the role of proteins corresponding to the candidate phosphosite signature according to available literature.

Since tyrosine kinase inhibitors such as sunitinib specifically target aberrant kinase signaling, a functional analysis of activated kinases is essential for a good understanding of sensitivity to sunitinib treatment. To this end, we performed INKA [24, 46–48] analysis to further explore the differences in tumor biology between individual sensitive and resistant patients. Overall, 51 unique tyrosine kinases were identified in 23 patients. For each patient, the top-20 most activated kinases were ranked (Additional file 4: Fig. S4). Mitogen-activated Protein Kinase (MAPK3) (p = 0.028) and EGFR (p = 0.045) showed significantly higher activity in sensitive patients compared to resistant patients. INSR/IGF1R was exclusively activated in a substantial number of sensitive patients (Fig. 1d). To gain further insight in the biological differences between the groups, a post-translational modifications (PTM) signature enrichment analysis (SEA) was performed. As opposed to gene set enrichment analysis (GSEA), PTM-SEA takes into account the specific combinations of sites of phosphorylation, making it more suitable for analyzing phosphoproteomics data. PTM-SEA showed that three phosphosite-centric signatures were significantly enriched (p < 0.05) in resistant patients: “FGF1 and prolactine pathways” and “EPHA substrates”. Fifteen signatures were enriched in sensitive patients, among which “insulin, VEGF and FGF2 treatment” and “KIT receptor pathway” (Fig. 1e).

### Proteome analysis

**Fig. 2:**
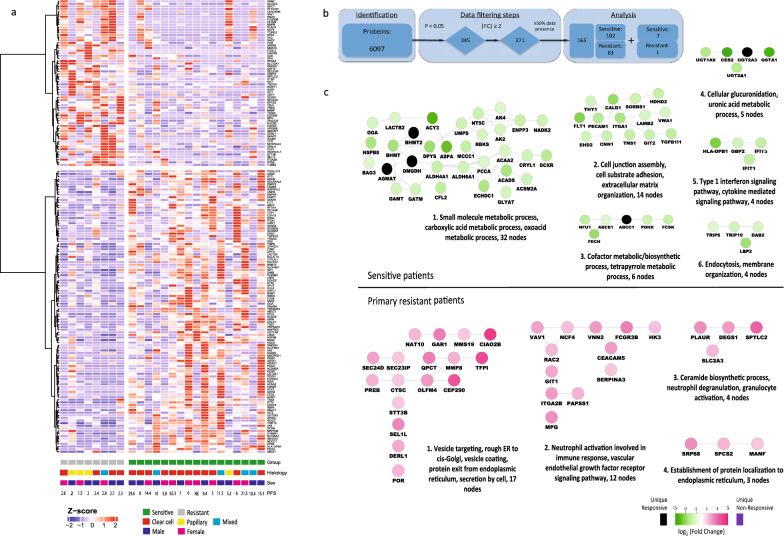
Proteome analysis of patients with RCC sensitive or resistant to sunitinib. Supervised clustering analysis of the proteome. **a** Supervised cluster analysis of differentially expressed proteins (*n* = 173) in tumor tissue lysates of 25 patients (17 sensitive and 8 resistant to sunitinib) shows one cluster of 13 sensitive patients and a mixed cluster of 8 resistant plus 4 sensitive patients. Filters: p < 0.05, |FC|> 2, ≥ 50% data presence in the highest group. For clustering, Euclidean distance and Ward’s linkage method were used. Histology = histological subtype as determined by pathologist review; PFS = progression free survival in months; NE = not evaluable. **b** Overview of the data filtering steps applied in protein analysis, including the effect of each filter on the total number. **c** Protein interaction networks. Using STRING and visualization in Cytoscape, major functional protein clusters, corresponding to either sensitive or resistant patients, are shown. Nodes correspond to upregulated proteins and edges symbolize physical or functional associations. Green clusters represent proteins upregulated in lysate of tumors sensitive to sunitinib and purple clusters represent proteins upregulated in lysate of tumors primary resistant to sunitinib. Representative GO terms identified by BiNGO analysis in both sensitive and resistant samples are listed together with the number of proteins (nodes) per cluster. All proteins in this figure are filtered for p < 0.05 & FC > 2 & ≥ 50% data presence in the group with highest abundance

Expression proteomics was successfully performed on lysate of 25 (17 sensitive and eight resistant) out of 26 samples. In total, 6097 unique proteins were identified (Additional file 8: Table S3), of which 173 were differentially expressed (p < 0.05 & FC > 2 & ≥ 50% data presence in group with highest abundance) (Fig. 2); 109 were upregulated in sensitive and 64 in resistant patients. Of these, FOSL2 was uniquely found in resistant tumors and seven proteins were unique in sensitive tumors (AGMAT, DMGDH, BHMT2, ABCC1, UGT2A3, MEM263 and RBP5). These 173 robust differential proteins are visualized in Fig. 2a, split by group. Gene ontology mining revealed that highly abundant proteins in resistant tumors were associated with vesicle mediated transport and excretion from cell processes, while in sensitive tumors, proteins with highest abundance were associated with multiple metabolic processes, such as small molecule -, carboxylic acid -, oxoacid—and glucoronate metabolic processes (Fig. 2c).

### Exploration of phospho-site and protein signature candidates in publicly available transcriptome data

To confirm our findings from this small cohort of patients, we searched the literature for a comparable independent cohort describing ideally phosphoproteome- or proteome-based profiles or an upstream RNA analysis in relation to clinical outcomes of patients treated with sunitinib. We were able to compare our findings to the results of a cohort by Beuselinck et al. describing the transcriptome in relation to sunitinib response [44]. Comparing five primary resistant patients to 43 sensitive patients in this independent cohort, 815 out of 17,561 transcripts were differentially expressed (p < 0.05) between the two groups. Thirty-six of the 173 differentially upregulated proteins in our analysis were also differentially upregulated at the RNA level in the independent cohort (3 in resistant (PLAUR, SLC2A3 and EIF4A1) and 33 in sensitive patients).

## Discussion

To our knowledge, this is the first combined mass spectrometry-based tyrosine-phosphoproteomics and expression proteomics analysis on tumor tissue from patients with advanced RCC in order to identify candidate predictive molecular biomarkers for treatment benefit of sunitinib. We report distinctive phosphosite and protein signatures and differential kinase and pathway activities that are associated with sensitive and resistant tumors.

Exploring the differences in biology between sensitive and resistant tumors, we first focused on the characteristics of primary resistant patients. In this group, 22 phosphosites were differentially upregulated, of which 4 phosphosites were uniquely identified in this group (BCAR3_Y117, EIF4A2_Y251, NOP58_Y272, GDI1_Y93) (Table 2). BCAR3 and GDI1 have a role in tumor development and progression and are correlated with resistance to systemic therapy in other tumor types, including breast cancer [28, 49–53]. EIF4A2 mutations are found in 0.7% of ccRCC [54], when found in other types of cancer, these mutations are associated with unfavorable prognosis and resistance to therapy [55, 56]. EIF4A2 is a highly homologous paralog of, and functionally indistinguishable from EIF4A1 [57], which was also differentially expressed in our cohort on the protein level and, in an independent study [44], on the RNA level. Interestingly, comparing tumor and normal adjacent ccRCC tissue samples, Li et al. report EIF4EBP1, another member of the translation initiation complex, as a downstream substrate of mTOR, and EIF4EBP1 phosphorylation was decreased in vitro by mTOR inhibition [58]. These four in resistant patients uniquely identified phosphosites have not previously been implied in RCC prognosis or prediction of sunitinib treatment outcome. Other differential phosphosites, yet non-uniquely upregulated in one of the groups, included STAT4_Y693 which is regulated upstream by TYK2, and ALOX5_Y95 which has a role in inflammatory processes [59, 60].

Looking further into the biology of primary resistant tumors by analyzing enriched phosphosite-centric signatures (PTM-SEA), we found that Fibroblast Growth Factor (FGF) 1 and PROLACTIN pathways and EPHA2 substrates were significantly enriched signatures (Fig. 1e). FGF is known to play a critical role in driving VEGF-independent tumor angiogenesis and FGFR signaling is an established resistance mechanism of VEGFR inhibition [61, 62]. Prolactin has been reported to be elevated in 45% of ccRCC patients [63], acting in a cytokine-like manner and as an important stimulatory regulator of the immune system. EPHA2 is overexpressed in renal cell carcinoma, associated with more advanced disease and angiogenesis [64] and has been implied as a mediator of sunitinib resistance in RCC [65].

On protein expression level, gene ontology mining of primary resistant tumors revealed that processes related to vesicle mediated transport and excretion were enriched (Fig. 2c). One could hypothesize that this possibly reflects enhanced ability of these tumors for drug efflux, contributing to drug resistance [66, 67]. Alternatively, this vesicle mediated transport may reflect activation of immune processes, for example degranulation of mast cells. This would corroborate our phosphoproteomics data, with post-translational modification signatures indicative of enhanced immune processes in resistant patients (Fig. 1e), which is in line with previously published work linking upregulation of cellular immune pathways and inflammatory markers to an unfavorable response to anti-VEGFR TKI’s in ccRCC [44, 68, 69].

Shifting our view towards the group of sensitive patients, we found a different biological profile. At the kinase level, INKA analysis showed significantly increased inferred kinase activity of MAPK3 and EGFR (Fig. 1d). EGFR is known for its activating effect on the MAPK signaling cascade [70]. Also the downstream substrates MAP2K1 and MAP2K2 were enriched in sensitive patients (Additional file 5: Fig. S5), pointing towards MAPK as a contributing signaling pathway in this group. In line with these findings, two MAPK1 sites (T185 and Y187) that are known to induce the activity of the MAPK pathway [71] were differentially phosphorylated in sensitive patients, as well as a uniquely identified EGFR site (Y1138) that is a known regulator of this pathway [72]. Several phosphorylated sites on different peptides identified in sensitive patients are being directly regulated by EGFR (PEAK1, EPHA2, TNK2, RPS27 and CAVIN1) [72], supporting EGFR activation in sensitive patients. Based on these results, we propose that EGFR-driven MAPK signaling plays an important role in sensitivity to sunitinib in RCC, and may present an alternative target for (combination) treatment [73]. This corroborates the findings of Li et al. who found their P3 phosphoproteomic subtype to be associated with the EGFR pathway and other kinases including MAPK3, that plays a role in VEGF/angiogenesis signaling [58]. PTM signatures associated with sunitinib sensitivity showed enrichment of VEGF, KIT, Thrombin signaling, vanadate and FGF2 treatment signatures (Fig. 1e), pointing towards the anti-angiogenic effects of sunitinib [74, 75].

Acknowledging the limited sample size of the sensitive (n = 16) and resistant (n = 7) tumors, our analyses may have been influenced by a number of other factors: (i) differences in pre-analytical handling of the frozen, archival specimen may have resulted in different cold ischemia times, potentially altering the phosphorylation profile [76, 77], (ii) the use of mostly primary tumor tissue, whereas treatment benefit is evaluated based on response of metastases and (iii) the range of intervals (median 6 months) between resection and start of systemic therapy may suggest indolent biology as a cause of longer PFS. However, we found no significant correlation between the time to start sunitinib and the PFS (Spearman’s rho -0.018). Also, the influence of longer storage time at − 80 °C of samples on the phosphorylation profile is unknown.

Our data are internally consistent based on reproducibly identified phosphosites and –peptides (see Fig. 1b and Additional file 2: Fig. S2b) as well as identified kinase-substrate relations (e.g. for INSR/IGF1R and INSULIN treatment; Fig. 1c, d). Lacking an external dataset, we have not been able to validate our 78-phosphosite candidate signature that may predict treatment outcome of sunitinib. For most (uniquely identified) differential phosphosites no antibodies were available for (technical) Western blot validation of the phosphoproteomic data. An exploratory comparison of our findings from the (phospho)proteomics analysis to transcriptome data as a proxy for (phospho)protein expression, using a comparable (n = 53) RCC cohort [44] showed limited overlap (36 of 173) between the differentially regulated proteins and transcripts. In addition to sample size as contributing factor, it is known that transcriptomic and (phospho)proteomic data provide different levels of biological information [23, 78, 79]. However, in resistant patients, three proteins/transcripts overlapped: PLAUR, SLC2A3 and EIF4A1. Interestingly, EIF4A1, a regulator of ERK signaling [80], was differentially upregulated on protein and transcript level, while its nearly identical homolog EIF4A2 was exclusively phosphorylated in resistant patients and represented in the candidate signature, stressing its potential importance in sunitinib resistance. Several identified differential kinases and substrates in our analysis show overlap with previous findings [23, 58], while some, such as WEE1 and BAP1, did not surface in our study. Although these kinases/substrates are important in RCC pathogenesis, they may not differ between sunitinib sensitive or resistant patients.

## Conclusions

This MS-based analysis of the RCC (tyrosine-phospho)proteome revealed disctinctive phosphosite and protein signatures and differential kinase and pathway activities that are associated with sunitinib sensitivity and resistance. One protein (EIF4A1 and its homolog EIF4A2) was confirmed to be differentially expressed on phosphosite, protein and RNA level. These findings warrant validation in an independent cohort and the clinical utility for treatment selection remains to be demonstrated. A targeted assay or immunohistochemistry analysis with a selection of differential phosphosites and/or proteins could facilitate the implementation of these signatures as a decision-making tool for treatment selection in clinical practice. Such an assay would prevent toxicity and enable alternative (combination) treatment in patients upfront predicted to be resistant to sunitinib.

### Supplementary Information

**Additional file 1: Figure S1.** Unsupervised cluster analysis of all detected phosphosites. After removal of non-human entries and phosphosites with only zero intensities measured, 1596 phosphosites in 23 samples were analyzed. Group based analysis using LIMMA statistics for differential phosphorylation. No imputation of data is performed. Euclidean distance and Ward’s linkage method were used. Histology = histological subtype as determined by pathologist review; *PFS* progression free survival in months, *NE* not evaluable.

**Additional file 2: Figure S2.** Phosphopeptide cluster analyses in sensitive and primary resistant patients. a Unsupervised cluster analysis of identified phosphopeptides. After removal of non-human entries and phosphopeptides with only zero intensities measured, 1900 phosphopeptides were analyzed. b Supervised cluster analysis of the differentially detected phosphopeptides (n=73) in sensitive and primary resistant patients. Non-unique phosphopeptides (n=24) are filtered for p <0.05, |FC| >2 and ≥30% data presence in the highest group. Unique phosphopeptides (n=49) are filtered for ≥30% data presence. Clustering is determined by non-unique phosphopeptides. No imputation of data is performed. Euclidean distance and Ward’s linkage method were used.

**Additional file 3: Figure S3.** Phosphosite interaction network of sensitive and resistant patients. Phosphosite (p-site) interaction network. Using STRING and visualization in Cytoscape, a functional p-site cluster is shown of differentially expressed and unique p-sites in sensitive and resistant patients. Nodes correspond to upregulated p-sites. Green nodes represent p-sites differentially upregulated in tumors sensitive to sunitinib (n=21) and black nodes represent p-sites uniquely identified in tumors sensitive to sunitinib (n=35). Pink nodes represent p-sites differentially upregulated in tumors resistant to sunitinib (n=18) and purple nodes represent p-sites uniquely identified in tumors resistant to sunitinib (n=4). The differential p-sites in this figure are filtered for p < 0.05 & |FC| > 2. The unique p-sites in this figure are filtered for ≥30% data presence in the group with highest abundance. The p-site MAPK1_Y187 is identified twice: once through quantification of a mono-phosphorylated peptide (FC = − 3.81) and once through quantification of a diphosphorylated peptide (FC = − 5.57).

**Additional file 4: Figure S4.** Ranking of most activated kinases per sample. Ranking of the top 20 active kinases (Y-axis) in tumors from 16 sensitive and 7 resistant patients. Bar graphs depict kinase ranking based on combined INKA scores of kinase- and substrate-centric analysis of tyrosine phosphoproteomics24. X-axis represents the INKA score for each kinase. Differentially activated kinases between the two groups (Figure 1c) are highlighted with dark (EGFR, MAPK3) and light (INSR/IGF1R) green coloring.

**Additional file 5: Figure S5.** Bar plots of activated kinase substrates in sensitive versus resistant patients. Activated kinase substrates that were enriched in sensitive patients (not significant), among which some of the known targets of sunitinib. X-axis: each bar represents a single patient (red = primary resistant, blue = sensitive), y-axis: INKA score of the kinase.

**Additional file 6: Table S1.** Clinicopathological data per individual patient.

**Additional file 7: Table S2.** All identified and quantified phosphosites.

**Additional file 8: Table S3.** All identified and quantified phosphopeptides and proteins.

**Additional file 9: Table S4.** Role of proteins corresponding to candidate phosphosite signature (n = 78) in RCC.

## Data Availability

The mass spectrometry proteomics data have been deposited to the ProteomeXchange Consortium via the PRIDE partner repository with the dataset identifier PXD043514.

## Abbreviations

(cc)RCC: (Clear cell) Renal cell carcinoma
DMEM: Dulbecco’s modified eagle medium
EGFR: Epidermal growth factor receptor
FBS: Fetal bovine serum
FC: Fold-change
FDR: False discovery rate
FGF: Fibroblast growth factor
GSEA: Gene set enrichment analysis
ICI: Immune checkpoint inhibitors
INKA: Integrative inferred kinase activity
IP: Immunoprecipitation
KIT: Stem cell factor receptor
LC–MS/MS: Liquid chromatography coupled to tandem mass spectrometry
MAPK: Mitogen-activated protein kinase
mRCC: Metastatic renal cell carcinoma
OS: Overall survival
PBS: Phosphate-buffered saline
PDGFR: Platelet-derived growth factor receptor
PFS: Progression-free survival
P-proteomics: Phosphoproteomics
PTM: Post-translational modification
PTM-SEA: Post-translational modifications signature enrichment analysis
pTyr: Phosphotyrosine
RES: Resistant
SENS: Sensitive
TKI: Tyrosine kinase inhibitor
VEGFR: Vascular endothelial growth factor receptor
