# Supplementary figures and images for: Candidate biomarkers for treatment benefit from sunitinib in patients with advanced renal cell carcinoma using mass spectrometry-based (phospho)proteomics
Source: Clin Proteomics. 2023 Nov 8;20:49. doi: 10.1186/s12014-023-09437-6 (PMC10631096; doi:10.1186/s12014-023-09437-6)

Additional Figure 1

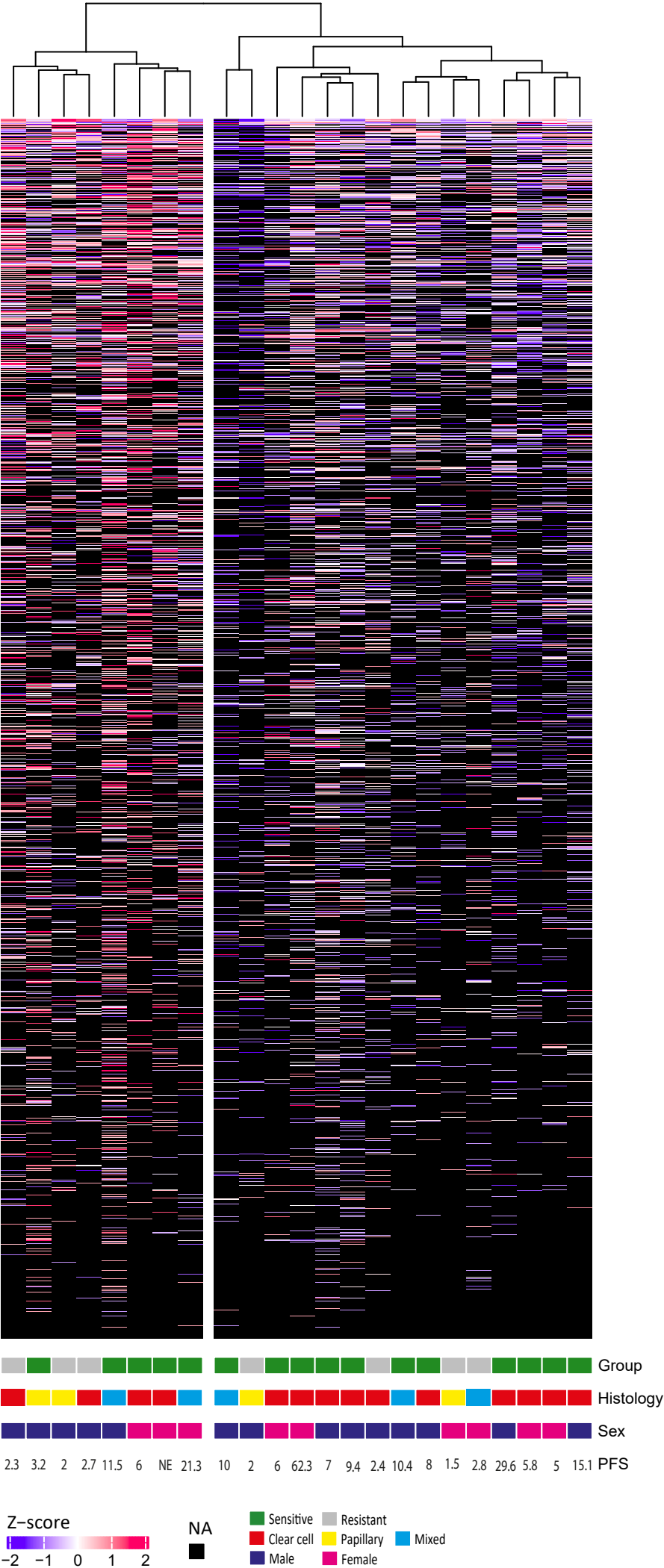

Supplement: Supplementary file 1 — Additional file 1: Figure S1. Unsupervised cluster analysis of all detected phosphosites. After removal of non-human entries and phosphosites with only zero intensities measured, 1596 phosphosites in 23 samples were analyzed. Group based analysis using LIMMA statistics for differential phosphorylation. No imputation of data is performed. Euclidean distance and Ward’s linkage method were used. Histology = histological subtype as determined by pathologist review; PFS progression free survival in months, NE not evaluable. [file 12014_2023_9437_MOESM1_ESM.pdf]

Additional Figure 2

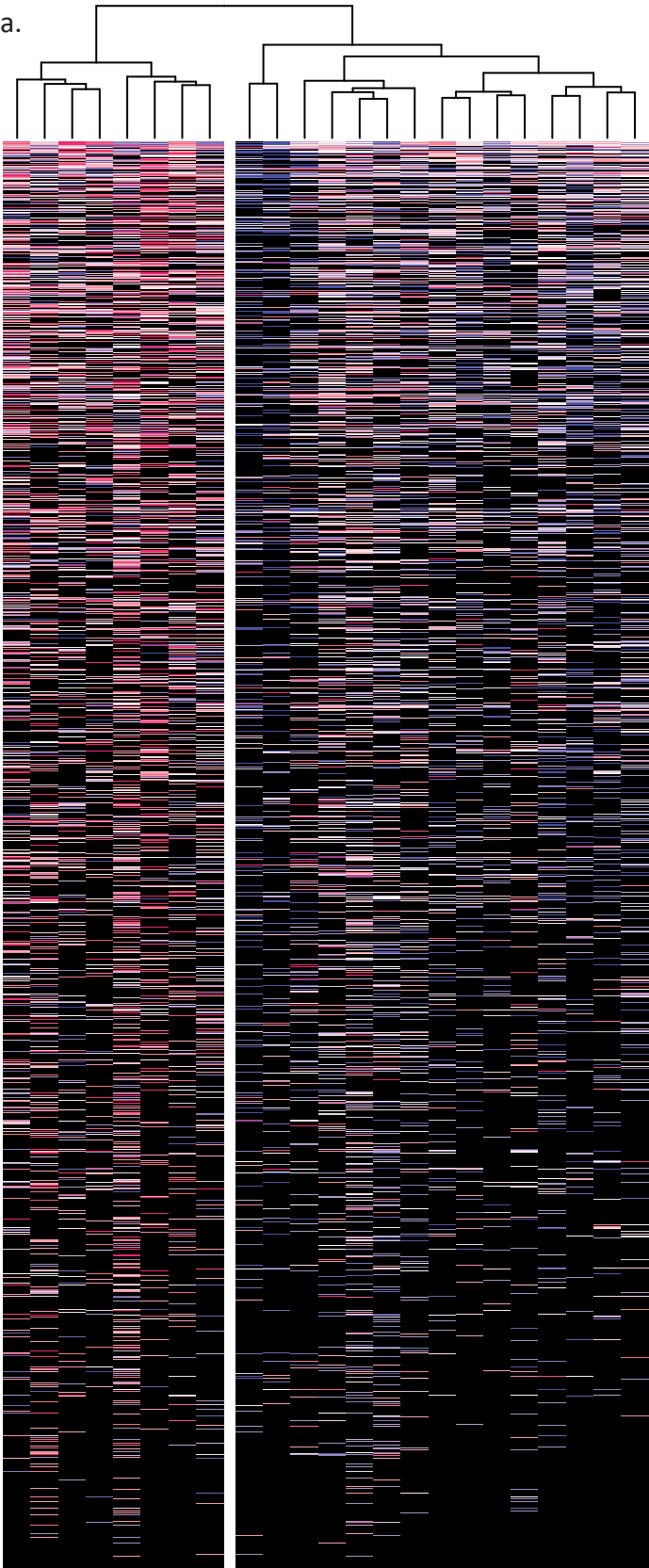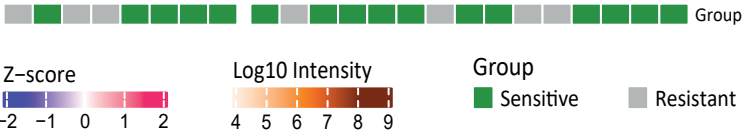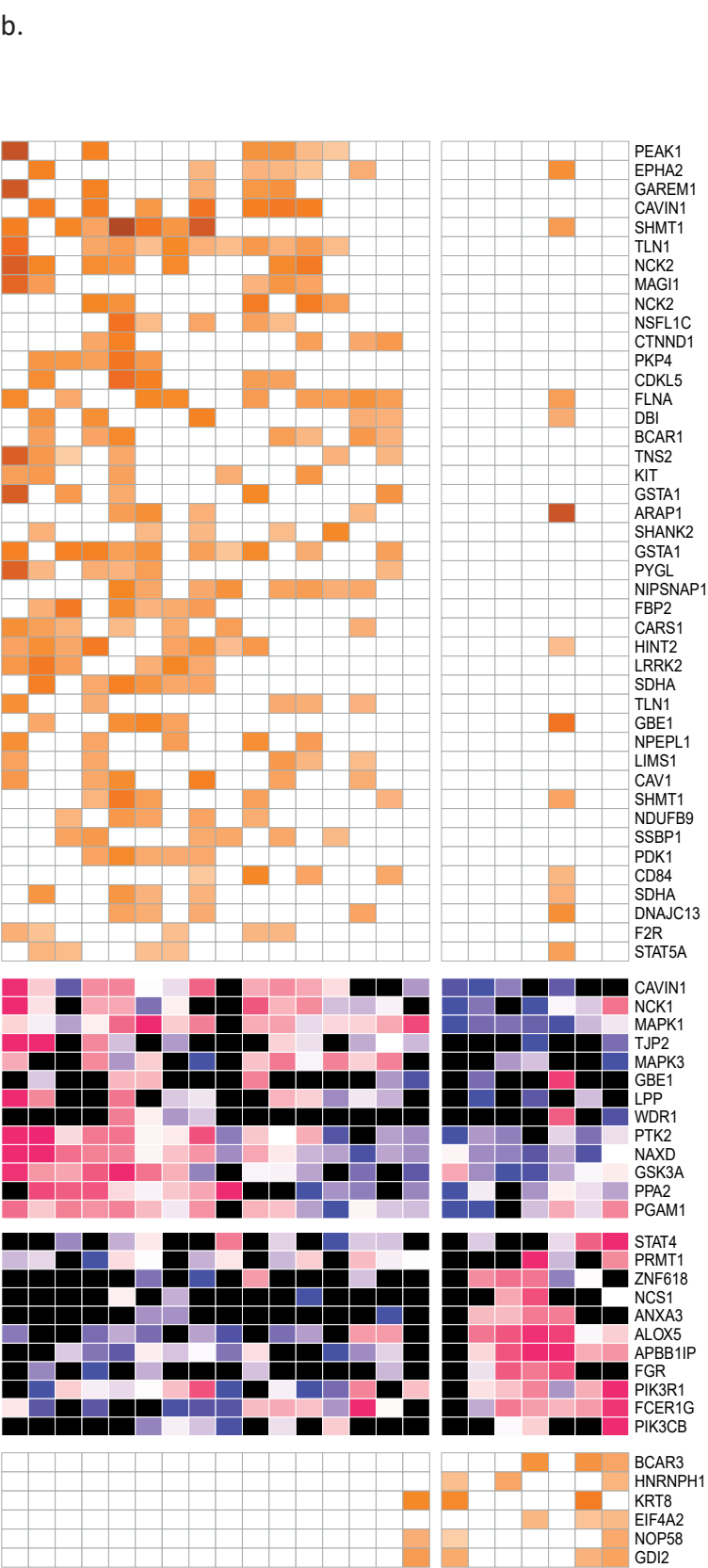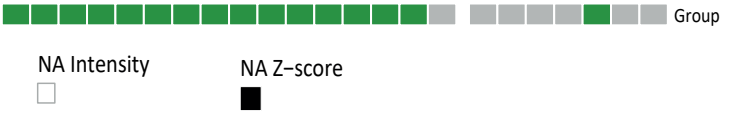

Supplement: Supplementary file 2 — Additional file 2: Figure S2. Phosphopeptide cluster analyses in sensitive and primary resistant patients. a Unsupervised cluster analysis of identified phosphopeptides. After removal of non-human entries and phosphopeptides with only zero intensities measured, 1900 phosphopeptides were analyzed. b Supervised cluster analysis of the differentially detected phosphopeptides (n=73) in sensitive and primary resistant patients. Non-unique phosphopeptides (n=24) are filtered for p <0.05, |FC| >2 and ≥30% data presence in the highest group. Unique phosphopeptides (n=49) are filtered for ≥30% data presence. Clustering is determined by non-unique phosphopeptides. No imputation of data is performed. Euclidean distance and Ward’s linkage method were used. [file 12014_2023_9437_MOESM2_ESM.pdf]

Additional Figure 3

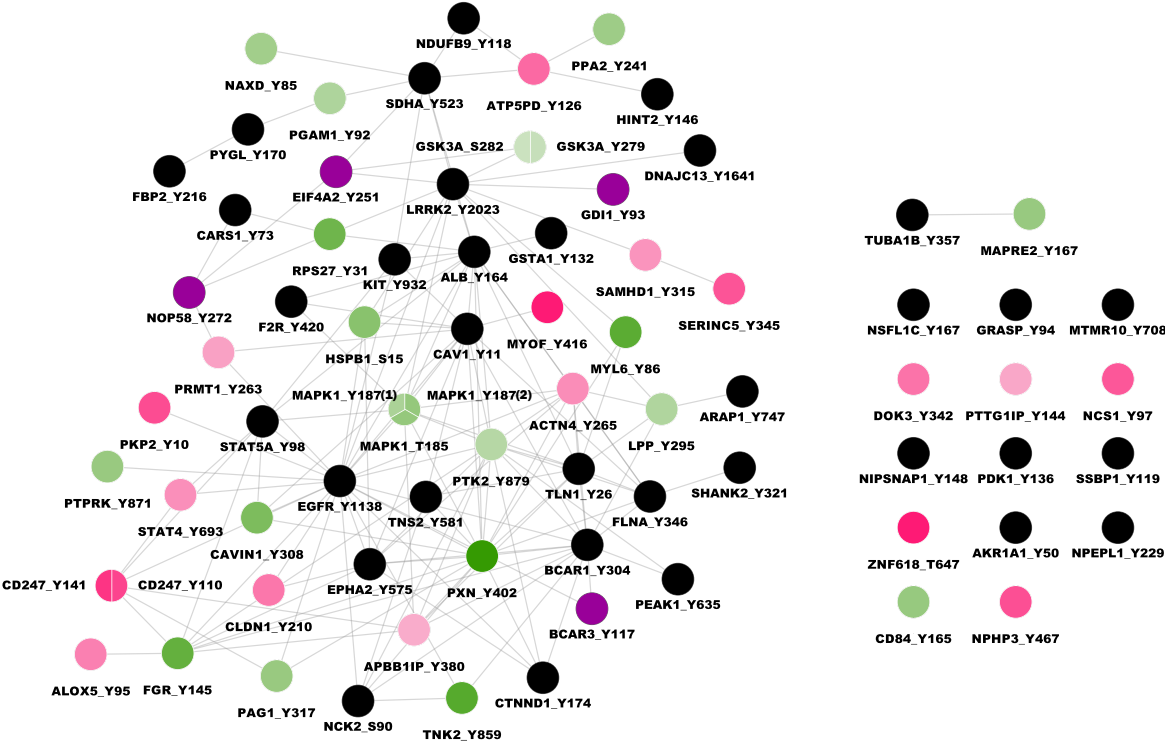

Supplement: Supplementary file 3 — Additional file 3: Figure S3. Phosphosite interaction network of sensitive and resistant patients. Phosphosite (p-site) interaction network. Using STRING and visualization in Cytoscape, a functional p-site cluster is shown of differentially expressed and unique p-sites in sensitive and resistant patients. Nodes correspond to upregulated p-sites. Green nodes represent p-sites differentially upregulated in tumors sensitive to sunitinib (n=21) and black nodes represent p-sites uniquely identified in tumors sensitive to sunitinib (n=35). Pink nodes represent p-sites differentially upregulated in tumors resistant to sunitinib (n=18) and purple nodes represent p-sites uniquely identified in tumors resistant to sunitinib (n=4). The differential p-sites in this figure are filtered for p < 0.05 & |FC| > 2. The unique p-sites in this figure are filtered for ≥30% data presence in the group with highest abundance. The p-site MAPK1_Y187 is identified twice: once through quantification of a mono-phosphorylated peptide (FC = − 3.81) and once through quantification of a diphosphorylated peptide (FC = − 5.57). [file 12014_2023_9437_MOESM3_ESM.pdf]

Additional Figure 5

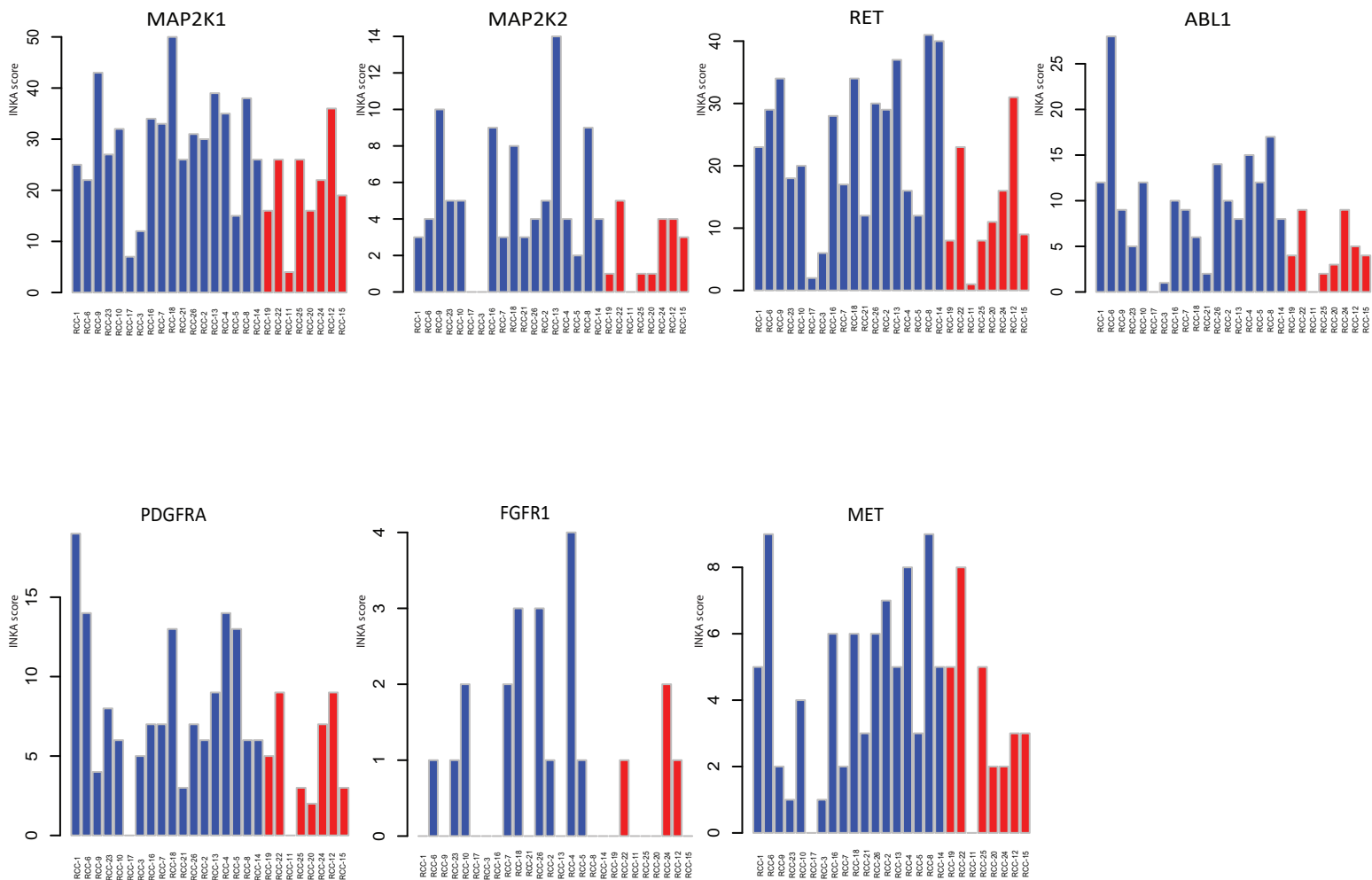

Supplement: Supplementary file 5 — Additional file 5: Figure S5. Bar plots of activated kinase substrates in sensitive versus resistant patients. Activated kinase substrates that were enriched in sensitive patients (not significant), among which some of the known targets of sunitinib. X-axis: each bar represents a single patient (red = primary resistant, blue = sensitive), y-axis: INKA score of the kinase. [file 12014_2023_9437_MOESM5_ESM.pdf]
