# Supplementary material for: Candidate biomarkers for treatment benefit from sunitinib in patients with advanced renal cell carcinoma using mass spectrometry-based (phospho)proteomics
Source: Clin Proteomics. 2023 Nov 8;20:49. doi: 10.1186/s12014-023-09437-6 (PMC10631096; doi:10.1186/s12014-023-09437-6)

Additional Figure 4: INKA rankings per patient - sensitive

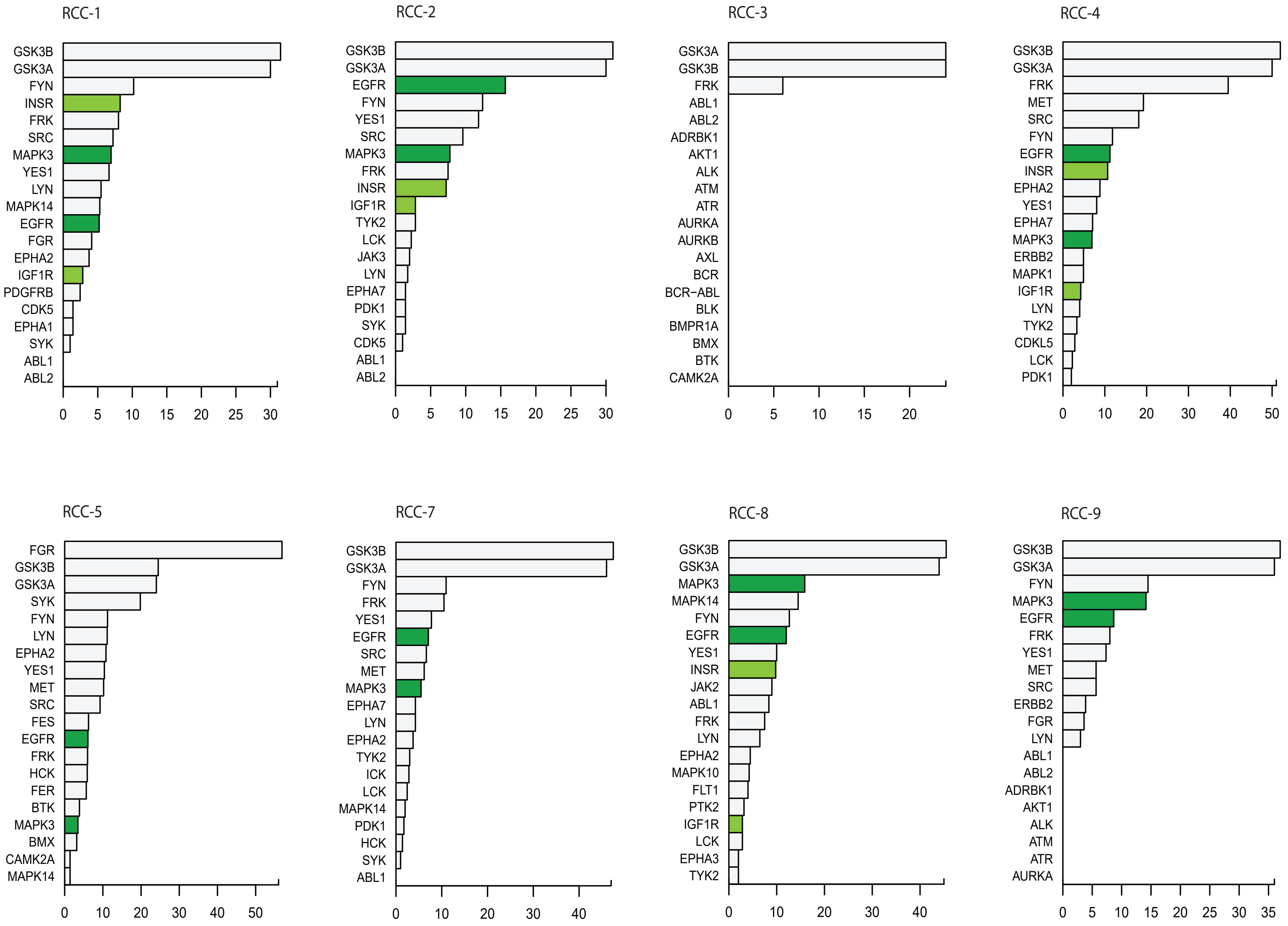

Additional Figure 4: INKA rankings per patient - sensitive

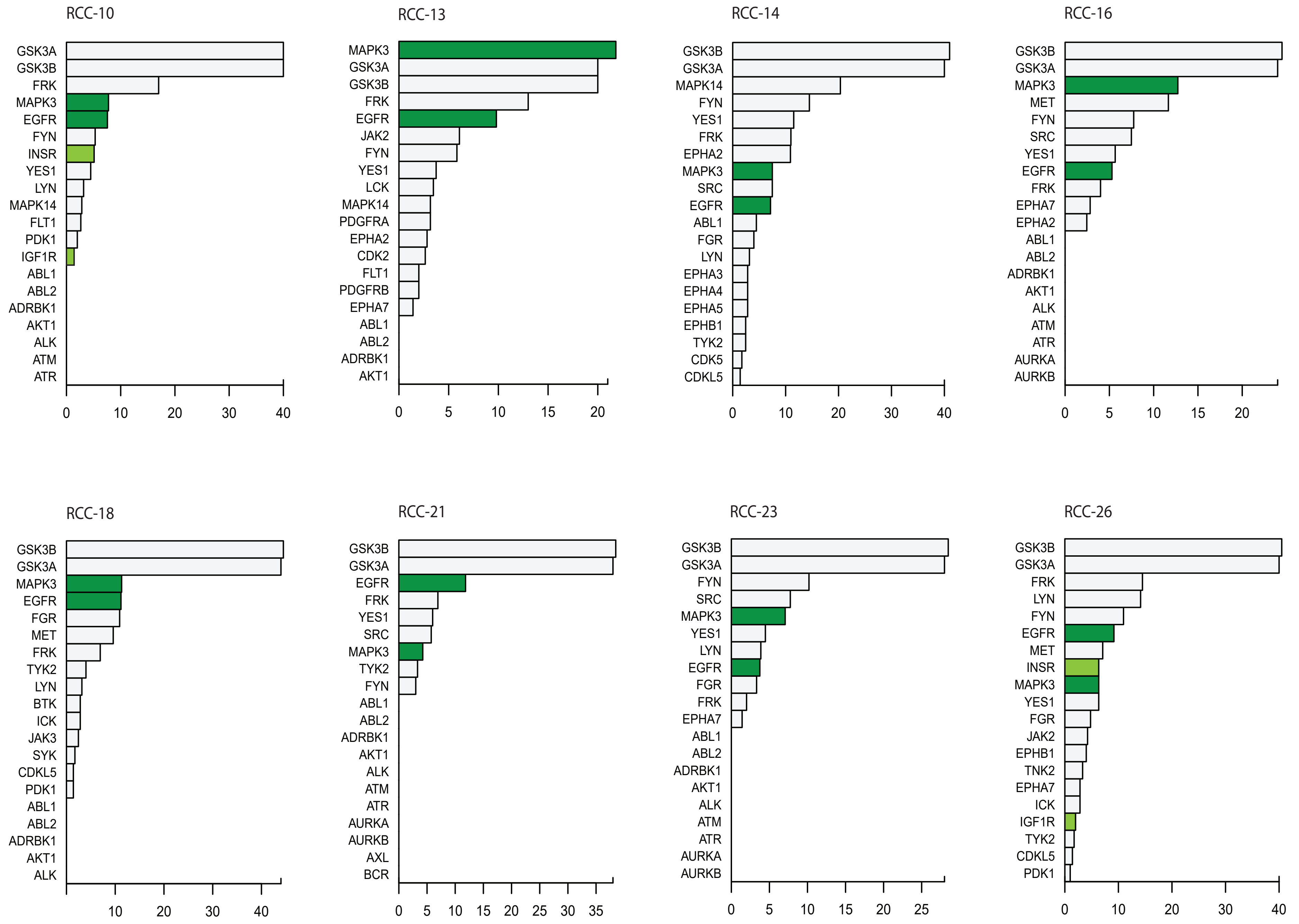

Additional Figure 4: INKA rankings per patient - primary resistant

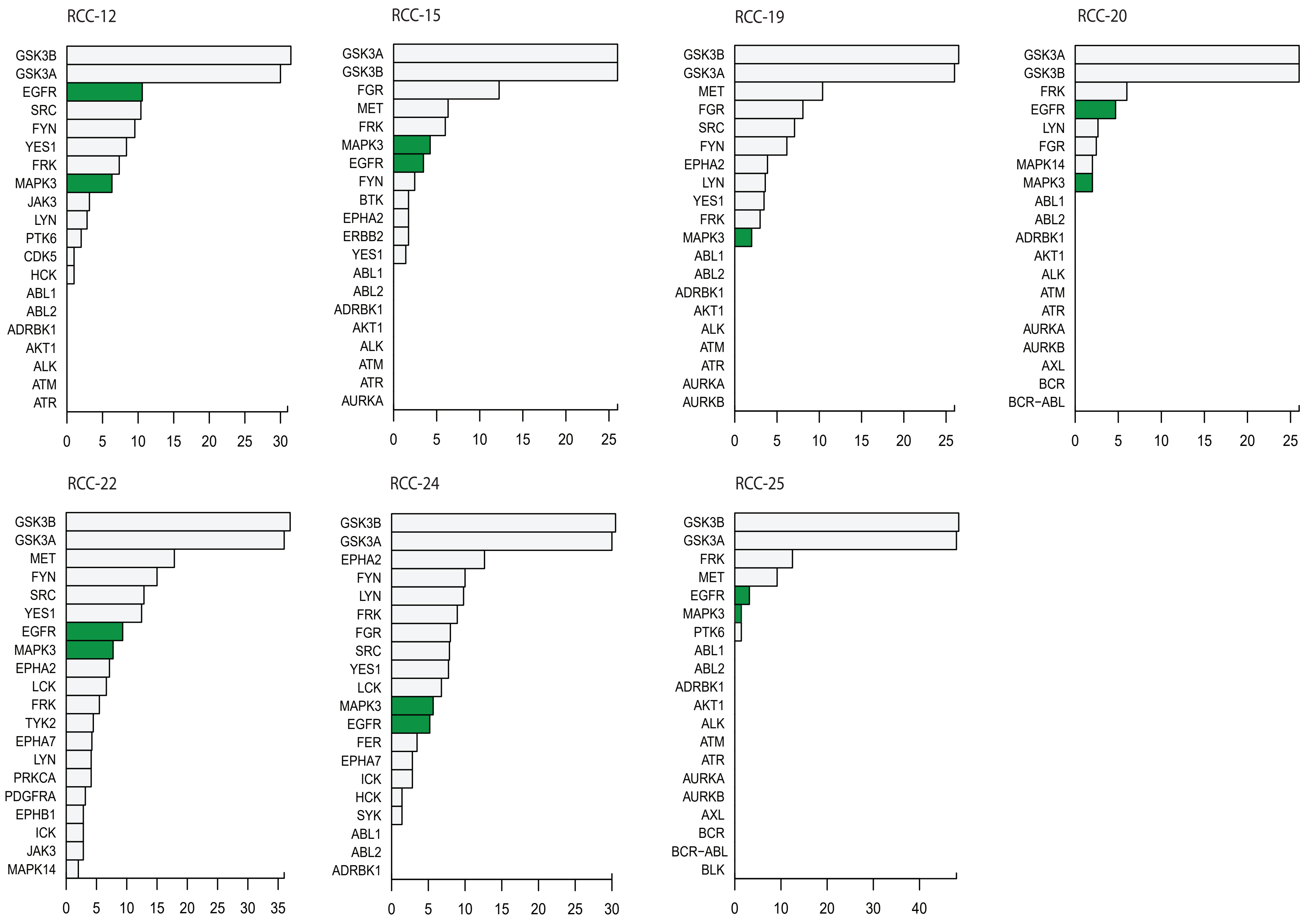

Supplement: Supplementary file 4 — Additional file 4: Figure S4. Ranking of most activated kinases per sample. Ranking of the top 20 active kinases (Y-axis) in tumors from 16 sensitive and 7 resistant patients. Bar graphs depict kinase ranking based on combined INKA scores of kinase- and substrate-centric analysis of tyrosine phosphoproteomics24. X-axis represents the INKA score for each kinase. Differentially activated kinases between the two groups (Figure 1c) are highlighted with dark (EGFR, MAPK3) and light (INSR/IGF1R) green coloring. [file 12014_2023_9437_MOESM4_ESM.pdf]
