# Supplementary material for: Candidate biomarkers for treatment benefit from sunitinib in patients with advanced renal cell carcinoma using mass spectrometry-based (phospho)proteomics
Source: Clin Proteomics. 2023 Nov 8;20:49. doi: 10.1186/s12014-023-09437-6 (PMC10631096; doi:10.1186/s12014-023-09437-6)
Supplement: Supplementary file 6 — Additional file 6: Table S1. Clinicopathological data per individual patient. [file 12014_2023_9437_MOESM6_ESM.docx]

## Additional Tables

### Additional Table 1: Clinicopathological data per individual patient

| Patient ID^1^ | Gender, age^2^ | Histology^3^ | Lesion | Prior immuno-therapy^4^ | Time to sunitinib^5^ | Best response^6^ | PFS^7^ | Tumor cell % | Protein input |
| --- | --- | --- | --- | --- | --- | --- | --- | --- | --- |
| RCC1 | M, 46 | CC | Primary | ASI trial | 7 | PR | 29.6 | 90 % | 5 mg |
| RCC2 | M, 59 | CC | Primary | ASI trial | 4 | PR | 9.4 | 90 % | 3 mg |
| RCC3 | M, 40 | AC / P | Primary | Interferon | 5 | SD | 10 | 80 % | 3 mg |
| RCC4 | M, 60 | AC / P | Primary | Interferon | 36 | SD | 11.5 | 60 % | 5 mg |
| RCC5 | M, 60 | P | Primary | None | 4 | SD | 3.2 | 40 % | 5 mg |
| RCC6 | M, 79 | CC / AC | Primary | None | 63 | PR | 9.5 | 80 % | 5 mg |
| RCC7 | F, 69 | CC | Primary | None | 2 | SD | 62.3 | 80 % | 5 mg |
| RCC8 | F, 60 | CC | Primary | None | 1 | SD/PR* | 6 | 80 % | 5 mg |
| RCC9 | M, 75 | CC | Primary | None | 10 | SD | 8 | 50 % | 5 mg |
| RCC10 | M, 66 | CC | Metastatic^8^ | None | 4 | SD | 15.1 | 90 % | 3 mg |
| RCC11 | F, 57 | CC | Primary | None | 2 | PD | 2.8 | NA | 5 mg |
| RCC12 | M, 64 | CC | Primary | None | 2 | PD | 2.4 | 90 % | 3 mg |
| RCC13 | F, 64 | CC | Primary | Interferon | 26 | SD | 5 | 90 % | 2 mg |
| RCC14 | F, 57 | CC / P | Primary | ASI trial | 5 | PR | 21.3 | 60 % | 5 mg |
| RCC15 | F, 42 | CC / E | Primary | None | 10 | PD | 2.8 | 25% | 5 mg |
| RCC16 | F, 62 | CC | Primary | ASI trial | 13 | PR | 5.8 | 50 % | 3 mg |
| RCC17 | F, 47 | CC | Primary | None | 10 | SD | 14.4 | NA | 5 mg |
| RCC18 | M, 69 | CC | Primary | None | 6 | SD | 7 | 80 % | 5 mg |
| RCC19 | M, 59 | CC | Primary | None | 4 | PD | 2.7 | 25 % | 5 mg |
| RCC20 | F, 20 | P | Metastatic^9^ | None | 1 | PD | 1.5 | 60 % | 5 mg |
| RCC21 | F, 67 | CC | Primary | Interferon | 15 | SD | 6 | 90 % | 5 mg |
| RCC22 | M, 54 | CC | Primary | ASI trial | 24 | PD | 2.3 | 80 % | 5 mg |
| RCC23 | M, 75 | CC / S | Primary | None | 1 | PR | 10.4 | 70 % | 3 mg |
| RCC24 | M, 80 | P | Primary | None | 8 | PD | 2 | 70 % | 5 mg |
| RCC25 | M, 80 | P | Primary | None | 16 | PD | 1.8 | 90 % | 5 mg |
| RCC26 | F, 53 | CC | Metastatic^10^ | None | 1 | SD | NE** | 90 % | 5 mg |

1: RCC = Renal Cell Carcinoma

2: Gender: M = male, F = female. Age = at start sunitinib

3: CC = clear cell, AC = adenocarcinoma, P = papillary, S = sarcomatoid, E = eosinophilic variant.

4: ASI trial^81^ = vaccination + CpG + GM-CSF, followed by CpG + Interferon

5: Time in months between nephrectomy or metastasectomy and start sunitinib.

6: Best overall response according to RECIST 1.1

7: Progression Free Survival in months

8: Metastatic site: lymph nodes in cavernous sinus

9: Metastatic site: liver

10: Metastatic site: local recurrence in renal fossa

*: Measurements of tumor target lesions could not be performed, scan was performed elsewhere, radiology report states “decrease of metastatic lesions”. Patient is considered as sensitive to sunitinib.

**: Metastatic lesion was resected after 2 months of therapy. Histological evaluation shows extensive necrosis of the metastatic lesion, therefore patient is considered as sensitive to sunitinib.

NA: not applicable. Due to extensive necrotic tumor tissue, the tumor cell percentage could not be determined.
