# Supplementary material for: Candidate biomarkers for treatment benefit from sunitinib in patients with advanced renal cell carcinoma using mass spectrometry-based (phospho)proteomics
Source: Clin Proteomics. 2023 Nov 8;20:49. doi: 10.1186/s12014-023-09437-6 (PMC10631096; doi:10.1186/s12014-023-09437-6)
Supplement: Supplementary file 9 — Additional file 9: Table S4. Role of proteins corresponding to candidate phosphosite signature (n = 78) in RCC. [file 12014_2023_9437_MOESM9_ESM.docx]

### Additional Table 4: Role of proteins corresponding to candidate phosphosite signature (n = 78) in RCC

Additional Table 4a: phosphosites upregulated in primary resistant patients

|  | Phosphosite | p-value | FC | Role of corresponding protein in RCC |
| --- | --- | --- | --- | --- |
| Uniquely upregulated in resistant tumors | BCAR3_Y117 | n/a | n/a | No literature describing a role in RCC. |
|  | EIF4A2_Y251 | n/a | n/a |  |
|  | NOP58_Y272 | n/a | n/a |  |
|  | GDI1_Y93 | n/a | n/a |  |
| Differentially upregulated (not unique) | ZNF618_T647 | 0.004 | 22.2 | No literature describing a role in RCC. |
|  | CD247_Y141 | 0.008 | 15.2 | A relatively high expression of CD247, which represents a target for immunotherapy, is found in ccRCC compared to normal tissue^82,83^ |
|  | MYOF_Y416 | 0.009 | 22.0 | MYOF influences cellular proliferation of the metastatic CCRCC cell line by regulating VEGFR2 degradation^84^ and MYOF hyperexpression was significantly associated with disease-free survival^85^. |
|  | CD247_Y110 | 0.013 | 12.2 | A relatively high expression of CD247, which represents a target for immunotherapy, is found in ccRCC compared to normal tissue^82,83^. |
|  | APBB1IP_Y380 | 0.018 | 2.8 | No literature describing a role in RCC. |
|  | PTTG1IP_Y144 | 0.018 | 3.1 | No literature describing a role in RCC. |
|  | ATP5PD_Y126 | 0.020 | 7.3 | ATP5PD is differentially expressed between ccRCC tissue en normal renal tissue^86^. |
|  | NCS1_Y97 | 0.022 | 9.4 | No literature describing a role in RCC. |
|  | DOK3_Y342 | 0.023 | 6.3 | In an *in vitro* study, DOK3 was downregulated in RCC cell lines after stimulation with insulin and insulin-like growth factors (IGFs), stimulating RCC tumorigenesis and progression^87^. |
|  | CLDN1_Y210 | 0.025 | 6.0 | CLDN1 is expressed in RCC cells in a PAX8-dependent manner^88^. |
|  | STAT4_Y693 | 0.029 | 4.2 | No literature describing a role in RCC. |
|  | PRMT1_Y263 | 0.030 | 3.3 | Expression may be characteristic for low grade and low stage ccRCC, whole homologous loss of PRMT1 may be significant for high grade and high stage ccRCC^89^. BTG1 may inhibit cell growth and promote apoptosis by interacting with PRMT1 in RCC^90^. |
|  | NPHP3_Y467 | 0.031 | 10.5 | No literature describing a role in RCC. |
|  | ALOX5_Y95 | 0.033 | 5.3 | Higher expression predicts reduced survival in ccRCC^91^. Upregulation of ALOX5 is an important step in RCC progression. VEGF expression was strongly inducible by ALOX5 metabolites in vitro^92^. ALOX5 inhibition causes marked reduction of RCC cells in vitro through apoptosis^93^. |
|  | PKP2_Y10 | 0.034 | 11.0 | PKP2 is a target gene and component of a protein network regulated by HIF2α and is associated with a poorer survival of patients with RCC^94^. |
|  | SERINC5_Y345 | 0.038 | 9.8 | No literature describing a role in RCC. |
|  | ACTN4_Y265 | 0.045 | 4.4 | No literature describing a role in RCC. |
|  | SAMHD1_Y315 | 0.047 | 3.9 | No literature describing a role in RCC. |

Additional Table 4b: phosphosites upregulated in sensitive patients

|  | Phosphosite | p-value | FC | Role of corresponding protein in RCC |
| --- | --- | --- | --- | --- |
| Uniquely upregulated in sensitive tumors | PEAK1_Y635 | n/a | n/a | No literature describing a role in RCC. |
|  | EPHA2_Y575 | n/a | n/a | Expression of EphA2 is positively associated with tumor size and Fuhrman nuclear grade in ccRCC^64^ and high expression is associated with poor disease outcome^95^. Enhanced YB1/EphA2 axis signaling promotes acquired resistance to sunitinib in RCC^65^. |
|  | NCK2_Y110 | n/a | n/a | No literature describing a role in RCC. |
|  | TLN1_Y26 | n/a | n/a | No literature describing a role in RCC. |
|  | EGFR_Y1138 | n/a | n/a | The epidermal growth factor receptor (EGFR) is overexpressed in RCC and it plays a critical role in tumorigenesis and progression in RCC^96-98^. EGFR hyperactivity in RCC is mediated by the VHL/HIF-2α/SMYD3 signaling cascade^99^. |
|  | CTNND1_Y174 | n/a | n/a | No literature describing a role in RCC. |
|  | CDK2_S90 | n/a | n/a | CDK2 kinase activity is required for proper cell cycle progression and is involved in oncogenesis of multiple tumor types, among which RCC^100^. |
|  | NSFL1C_Y167 | n/a | n/a | No literature describing a role in RCC. |
|  | FLNA_Y346 | n/a | n/a | FLNA expression is correlated with lymph node metastases, clinical stage, histological grade and poor overall survival in RCC, suggesting that it plays a role as tumor suppressor in RCC^101^. |
|  | MTMR10_Y708 | n/a | n/a | No literature describing a role in RCC. |
|  | AKR1A1_Y50 | n/a | n/a | No literature describing a role in RCC. |
|  | BCAR1_Y304 | n/a | n/a | No literature describing a role in RCC. |
|  | GRASP_Y94 | n/a | n/a | No literature describing a role in RCC. |
|  | TUBA1B_Y357 | n/a | n/a | No literature describing a role in RCC. |
|  | TNS2_Y581 | n/a | n/a | No literature describing a role in RCC. |
|  | ARAP1_Y747 | n/a | n/a | No literature describing a role in RCC. |
|  | SHANK2_Y321 | n/a | n/a | No literature describing a role in RCC. |
|  | GSTA1_Y132 | n/a | n/a | The exosomal shuttle RNA GSTA1 was significantly decreased in the urinary extracellular vesicles of patients with ccRCC compared to healthy subjects^102^. |
|  | PYGL_Y170 | n/a | n/a | No literature describing a role in RCC. |
|  | NIPSNAP1_Y148 | n/a | n/a | No literature describing a role in RCC. |
|  | SDHA_Y523 | n/a | n/a | SDHA is one of the four subunits of SDH, a well-recognized tumor suppressor gene involved in RCC carcinogenesis by SDH deficiency-driven HIF-α stabilization and activation, leading to increased VEGF-mediated angiogenesis. SDH deficient RCC form a distinct clinicopathological subtype of RCC^103^. |
|  | FBP2_Y216 | n/a | n/a | No literature describing a role in RCC. |
|  | HINT2_Y146 | n/a | n/a | No literature describing a role in RCC. |
|  | KIT_Y932 | n/a | n/a | The c-KIT receptor is activated by its ligand stem cell factor (SCF) and induces several signal transduction pathways (MAPK, PI3K, AKT) and leads to mast cell activation and secretion of pro-angiogenic cytokines. In RCC, the c-KIT receptor activation induces cross-talk between cancer cells, endothelial cells and mast cells, leading to strengthening of pro-angiogenic signaling^104-106^. c-KIT receptor is one of the main targets of the multi-kinase inhibitor sunitinib. |
|  | LRRK2_Y2023 | n/a | n/a | LRRK2 amplification increases MET signaling activation and promotes efficient tumor cell growth and survival in papillary renal cell cancer^107^. |
|  | CARS1_Y73 | n/a | n/a | No literature describing a role in RCC. |
|  | ALB_Y164 | n/a | n/a | A decreased pretreatment serum albumin (ALB) level implies a poor prognosis in RCC patients, with a worse progression free and overall survival^108^. |
|  | NPEPL_Y229 | n/a | n/a | No literature describing a role in RCC. |
|  | CAV1_Y11 | n/a | n/a | CAV1 interacts with the EGFR/RAS/ERK and PI3K/AKT pathways and promotes cell invasion, cell growth and VEGF-A secretion^109^. |
|  | NDUFB9_Y118 | n/a | n/a | Seven subunits of the mitochondrial complex I, among which NDUFB9, had downregulated mRNA expression in ccRCC^110^. |
|  | SSBP1_Y119 | n/a | n/a | No literature describing a role in RCC. |
|  | PDK1_Y136 | n/a | n/a | PDK1 mRNA expression is upregulated in RCC compared to normal tissue and is negatively correlated with tumor stage^111^. In vitro, low expression of PDK1 inhibits proliferation, migration and epithelial mesenchymal transition in RCC^112^. |
|  | DNAJC13_Y1641 | n/a | n/a | No literature describing a role in RCC. |
|  | F2R_Y420 | n/a | n/a | No literature describing a role in RCC. |
|  | STAT5A_Y98 | n/a | n/a | Dihydrotestosterone promotes cell proliferation through STAT5 activation in RCC cells^113^. |
| Differentially upregulated (not unique) | HSPB1_S15 | 0.001 | -6.9 | HSPB1 (=HSP27) is significantly overexpressed in RCC compared to normal kidney tissue^114^ and when activated, attributes to promotion of cancer development and metastatic potential. Inactivation of the pathway may attenuate the invasion and migration capabilities in RCC^115^. |
|  | PPA2_Y241 | 0.004 | -4.8 | No literature describing a role in RCC. |
|  | CAVIN1_Y308 | 0.005 | -8.6 | CAVIN1/PTRF expression in ccRCC is regulated by SHC1 through the EGFR pathway. Abnormal PTRF, which is detected in exosomes from urine, could be a potential marker for ccRCC diagnosis and treatment^116^. |
|  | PTK2_Y879 | 0.007 | -3.1 | Focal Adhesion Kinase (FAK/PTK2) is constitutively expressed in RCC and has a contributing role in proliferation, migration and invasion^117^. |
|  | MYL6_Y86 | 0.009 | -15.5 | No literature describing a role in RCC. |
|  | NAXD_Y85 | 0.012 | -4.5 | No literature describing a role in RCC. |
|  | MAPK1_Y187 | 0.015 | -3.8 | Constitutive activation of the MAPK signaling cascade plays a crucial role in tumorigenesis and metastasis in RCC^118^. MAPK1 (=ERK2) is significantly overexpressed in RCC compared to normal tissue^119^. |
|  | GSK3A_Y279 | 0.023 | -2.3 | No literature describing a role in RCC. |
|  | TNK2_Y859 | 0.027 | -16.9 | A mutation in the ACK1 (= TNK2) ubiquitin associated domain enhances oncogenic signaling through EGFR regulation in RCC cells^120^. |
|  | LPP_Y295 | 0.028 | -3.4 | No literature describing a role in RCC. |
|  | PXN_Y402 | 0.031 | -30.6 | The mRNA expression of paxillin (PXN) was upregulated in metastatic RCC cells compared to normal renal tissue. Paxillin upregulation may contribute to the pathogenicity and/or metastatic propensity of RCC and may have a role as potential marker of metastasis in RCC cells^121^. |
|  | FGR_Y145 | 0.032 | -13.4 | FGR is one of the five highly expressed Src family kinases in ccRCC. A relation with survival or response to therapy has not been demonstrated^122^. |
|  | GSK3A_S282 | 0.036 | -2.2 | No literature describing a role in RCC. |
|  | RPS27_Y31 | 0.038 | -11.0 | No literature describing a role in RCC. |
|  | MAPRE2_Y167 | 0.040 | -5.3 | No literature describing a role in RCC. |
|  | MAPK1_Y187 | 0.041 | -5.6 | Constitutive activation of the MAPK signaling cascade plays a crucial role in tumorigenesis and metastasis in RCC^118^. MAPK1 (=ERK2) is significantly overexpressed in RCC compared to normal tissue^119^. |
|  | MAPK1_T185 | 0.041 | -5.6 | Constitutive activation of the MAPK signaling cascade plays a crucial role in tumorigenesis and metastasis in RCC^118^. MAPK1 (=ERK2) is significantly overexpressed in RCC compared to normal tissue^119^. |
|  | PAG1_Y317 | 0.042 | -5.3 | Overexpression of Csk-binding protein (= PAG1) is found in over 70% of RCC tissues and contributes to renal cell carcinogenesis^123^. |
|  | PTPRK_Y871 | 0.042 | -5.3 | No literature describing a role in RCC. |
|  | PGAM1_Y92 | 0.042 | -3.6 | PGAM1 is highly expressed in ccRCC and correlates with clinicopathological features, such as tumor size^124^. |
|  | CD84_Y165 | 0.044 | -5.4 | No literature describing a role in RCC. |
